# Sound Absorption Performance of Ultralight Honeycomb Sandwich Panels Filled with “Network” Fibers—*Juncus effusus*

**DOI:** 10.3390/polym16131953

**Published:** 2024-07-08

**Authors:** Zhao Liu, Chenhao Dong, Lu Tong, Chris Rudd, Xiaosu Yi, Xiaoling Liu

**Affiliations:** 1 Faculty of Science and Engineering, The University of Nottingham Ningbo China, Ningbo 315100, China; zhao.liu@nottingham.edu.cn (Z.L.);; 2 James Cook University Singapore, 149 Sims Drive, Singapore 387380, Singapore

**Keywords:** *Juncus effusus*, sound absorption, honeycomb, sandwich panels, artificial neural network

## Abstract

This study investigates lightweight and efficient candidates for sound absorption to address the growing demand for sustainable and eco-friendly materials in noise attenuation. *Juncus effusus* (JE) is a natural fiber known for its unique three-dimensional network, providing a viable and sustainable filler for enhanced sound absorption in honeycomb panels. Microperforated-panel (MPP) honeycomb absorbers incorporating JE fillers were fabricated and designed, focusing on optimizing the absorber designs by varying JE filler densities, geometrical arrangements, and MPP parameters. At optimal filling densities, the MPP-type honeycomb structures filled with JE fibers achieved high noise reduction coefficients (NRC) of 0.5 and 0.7 at 20 mm and 50 mm thicknesses, respectively. Using an analytical model and an artificial neural network (ANN) model, the sound absorption characteristics of these absorbers were successfully predicted. This study demonstrates the potential of JE fibers in improving noise mitigation strategies across different industries, offering more sustainable and efficient solutions for construction and transportation.

## 1. Introduction

It is presently estimated that the industrial market for sound absorption materials is dominated by porous structures manufactured from petroleum-based polymers (polyurethane foams and expanded/extruded polystyrene foams) and rock- and slag-based fibers (glass wools and mineral wools); the market share of these materials is over 90%. As a result, these synthetic porous materials are derived from non-renewable resources and generate emissions during their manufacturing. The present study aims to identify green and sustainable media for sound attenuation applications [1,2,3,4,5,6]. Bio-sourced raw materials can reduce energy consumption, emissions, and convenient degradation after use (i.e., composting). They also can act as carbon sinks during the growing cycle [7]. Natural fibers for cordage, textiles, and structural products are an excellent example of this genre. Vegetable, animal, and mineral fibers, such as stalk fiber like rice and hardwood; skin fiber like jute, flax, and hemp; fruit fiber like coconut fiber; and animal fibers like chicken feathers and sheep wool have all been examined for the replacement of common synthetic fibers.

Noise pollution, caused by rapid urbanization, and industrialization, especially in Asian countries, is currently a major public health problem. Currently, the solutions to these problems are based on porous and resonant materials such as glass wool [8,9,10]. Whilst these are all effective absorbers of noise, their dumping in landfill sites at the end of their life impacts the natural environment. Recently, some attention has been paid to developing bio-sourced and sustainable sound absorbers. Previous research has utilized kenaf, jute, bamboo, yucca, milkweed, coir, date palm, and bagasse, which have useful potential for commercial applications. Natural fibers are thus applied in acoustic mufflers as fiber networks and composites based on natural fibers. Berardi et al. [11] showed that kenaf fibers provided good sound absorption at 200–2000 Hz. They also found that coconut fibers perform at low and medium frequency ranges. The influence of density, thickness, and air gap on the sound absorption of pineapple-leaf fibers was studied and researched by Putra et al. [12] The sound absorption performances at the medium frequency with 30 mm thickness and at the high-frequency range with 20 mm were excellent. Samaei et al. [13] developed a fibro-granular sound-absorbing composite using kenaf in fiber form and cylindrical rice husk as a natural granule. The sound absorption coefficient of this composite was over 0.8 at 1000 Hz, obtained ideal sound absorption at 1500 Hz, and maintained a high-level sound absorption at high frequencies. These natural fibers and their products are useful in acoustic attenuation, especially at medium and high frequencies.

*Juncus effusus* (JE) (common or corkscrew rush) is native to many temperate and tropical regions. It is a familiar feature of wetlands, found in many Asian countries with some traditional medicine applications, and widely used for matting products. Its porous structure resembles natural fibers comprising cellulose, hemicellulose, and lignin. However, its morphology is distinctive, resembling a three-dimensional foam with smaller pores at the micrometer level. JE fibers are typically slender cylinders with a 2–3 mm diameter and a length of up to 120 cm. Moreover, they possess porous and three-dimensional network structures, forming natural porous structures. These unique characteristics have been applied as industrial absorbents, e.g., cigarette filters [14], oil spill absorbents [15], dyestuff adsorbents for wastewater [16], and electrochemistry [17]. According to the sound-absorbing mechanism of porous absorbers, the spongy characteristics may also be beneficial to sound absorption but are seldom reported.

In research on honeycomb sound absorbers, the quest to elevate the acoustic efficacies of honeycomb structures often stems from their advantages in terms of comprehensive rigidity and damping capacities [18,19]. Yang [20] et al. engineered honeycomb sandwich panels incorporating glass fibers as fillers, targeting sound absorption and insulation investigations. This research considered variables such as the filler shape, fiber diameter, content, and presence of an air layer, which significantly influence the material’s sound insulation performance yet have a marginal effect on sound absorption. Xie [21] et al. developed an innovative composite structure by merging Nomex^®^ honeycomb with polyester fibers as fillers. Our previous research also involved various fibers as fillers for sound absorption properties. In our early studies of pre-screen fillings in honeycomb structures [22], we observed that the permeability of the honeycomb significantly influences the overall sound absorption efficiency. Earlier samples with densities ranging from 0.14 to 0.27 g/cm^3^ demonstrated promising lightweight performance. The choice of JE fibers, with a true density of approximately 0.36 g/cm^3^, is motivated by their potential to optimize this lightweight performance further, leveraging their lighter characteristics and more efficient sound absorption ability.

In this work, the intrinsic sound absorption properties of JE fibers and their effectiveness as a filling material in MPP-type honeycombs were studied. Firstly, the effects of different densities and geometric arrangements of JE fibers on sound absorption performance were studied. Secondly, the influences of various parameters of MPP on sound absorption performance were researched. Third, analytic and machine learning models were used to predict the sound absorption performance of MPP-type honeycomb absorbers. Based on those above, this structure demonstrates significant potential in weight reduction, good absorption, and mitigation of associated process pollutants.

## 2. Materials and Methods

### 2.1. Materials

**Figure 1:**
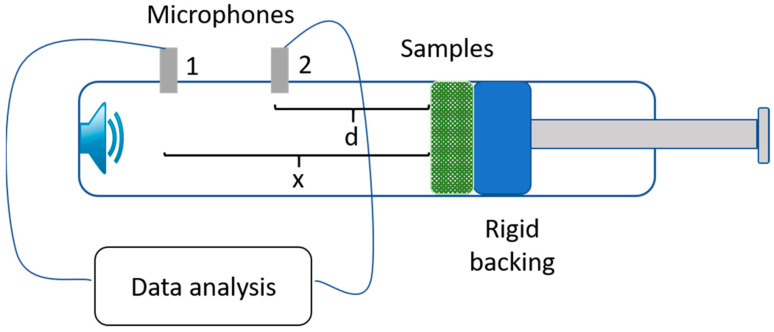
The schematic image of impedance tube.

**Figure 2:**
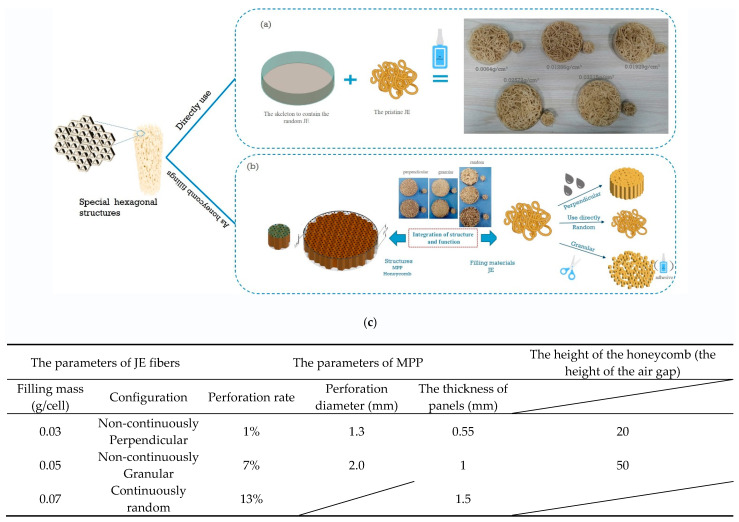
(**a**) The schematic image of the JE sample as acoustic absorbers and the manufacturing process; (**b**) the schematic image of the JE fibers as the fillings in honeycomb structures and the manufacturing process; (**c**) parameter table for JE fibers as fillers for MPP as face sheets in honeycomb structures.

Commercial Nomex^®^ honeycomb was purchased from CMAG Composite Co., Ltd., Jiaxing, China, with a density of 40.15 ± 4.0 kg/m^3^ as the containment for the acoustic media. The side length of the hexagonal cell was 5.5 mm with a height of 20 mm and 50 mm. Microperforated panels as face sheets made from polymethyl methacrylate (PMMA) were bought in Shenzhou Company, Foshan, China. The parameters of mechanically perforated holes on panels are shown in Figure 2c. Juncus effuses were bought from Primary Agricultural Chinese Medicinal Materials in Bozhou, Anhui province, China. The perpendicular and granular particles (Figure 1) were pretreated before filling into the honeycomb cell. The pristine perpendicular JE straightened after soaking with tap water and sectioned into 20 mm columns. The granular JE particles were cut in a juicer cup (Jiuyang Company, Jinan, China) with deionized water as lubricants and then dried up. Dry Lay-up Adhesive from 3M was used to assemble the sandwich panels. 

### 2.2. Characteristics and the Sound Absorption Experiment

The morphologies of JE fibers were observed by scanning electron microscope (SEM, SIGMA/VP, Zeiss, Germany) under 5 kV. Optical images were examined using an optical microscope (UM016) from Mustech Electronics Co., Ltd. (Shenzhen, China) The true density of JE fibers was obtained in a gas displacement pycnometer system (Ultrapyc 5000) from Anton Paar Co., Ltd., Graz, Austria).

The sound absorption coefficient of normal incidence was measured using a BSWA impedance tube (Beijing, China) according to ASTM E1050. The high- and low-frequency tests were conducted in different impedance tubes, with larger tubes for low frequency. The 63–1600 Hz tests were performed in a 100 mm diameter tube (SW422) and the 1000–6300 Hz tests were conducted in a 30 mm tube (SW477). The test frequency span was 2 Hz and testing was performed at room temperature. The transfer function method is applied in calculating the sound absorption coefficient, when the sound pressure measured by microphone 1 and 2 and the transfer function H_12_ can be obtained. The reflection coefficient can be obtained through the formula, k_0_ represents the complex wave number, d means the distance of the sample surface and microphone 2, and x means the distance between microphone 1 to the sample surface.

(1) $$R=\frac{H_{12}-e^{-jk_{0}d}}{e^{jk_{0}d}-H_{12}}e^{j2k_{0}x},$$

(2) $$\alpha =1-{\left|R\right|}^{2}$$

For sound absorption testing, each sample underwent three parallel tests to ensure data reproducibility.

The measurements of the air permeability of honeycomb with JE fibers as filings were conducted by the YG461E digital fabric air permeability meter manufactured by Wenzhou Baien Instrument Co., Ltd. (Wenzhou, China), according to ISO 9273:1995. For air permeability testing, more than ten tests were conducted at different positions for each sample group.

### 2.3. Manufacturing of the Acoustic Absorption Sample

Figure 2a shows the manufacturing process of the JE absorbers prior to the combination with MPPs and honeycomb. A PVC cylinder with a polymethyl methacrylate base was used as a sample holder for the initial sound absorption tests. These samples are used to test the sound absorption performance of JE fibers only. The filling masses of these samples are 1, 2, 3, 4, and 5 g, respectively, to achieve the corresponding densities. Figure 2b illustrates the assembly of MPP-type honeycomb structures with JE fillings. These samples consisted of microperforated panels (MPP) and honeycomb, with different groups of JE fibers filled in the honeycomb cell. The perpendicular particles were soaked in water, allowed to straighten, and then sectioned into 20 mm columns to adapt to the honeycomb cells. The random JE fibers were stuffed directly and crammed into each cell with a pre-weighed charge. The granular particles were made from pristine JE fibers through random cuts. The film adhesive was subsequently added at a little loading and cured at room temperature. Figure 2c summarizes the variables of JE fibers and the MPPs.

### 2.4. The Calculation of Porosity 

The porosity of materials is important to influence the sound absorption performance and the true density and the filling density of materials, as below, determine the porosity:

(3) $$Porosity=1-\frac{\rho_{s}}{\rho_{f}}$$

where *ρ_s_* represents the density of filling materials and *ρ*_f_ represents the true density of the fibers measured by the equipment

### 2.5. Artificial Neural Network Model

**Figure 3:**
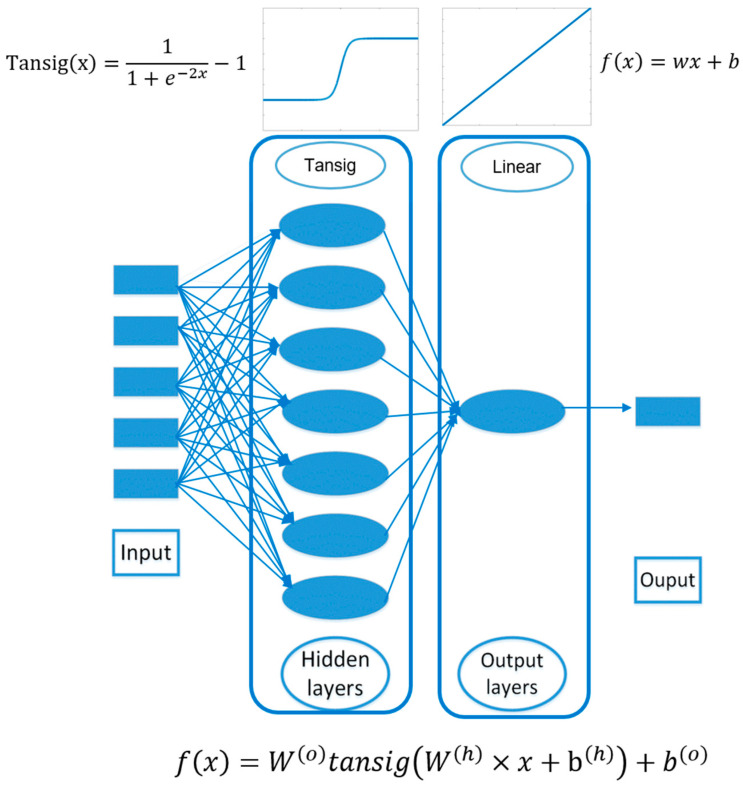
The three-layer algorithmic structure of the ANN model for sound absorption coefficient prediction.

An artificial neuron is a computational device that can generate an output signal based on a specific number of input signals that it receives. When input signals are received by an artificial neuron, they are passed through the activation function, which determines the specific output that the neuron will generate. The activation function operates by assigning a weight to each input signal and then adding them together. The activation function may also consider the value of the previous output signal of the neuron before the introduction of new signals. After obtaining the value from the activation function, it is then passed to the transfer function, which is responsible for generating the output signal. Neurons in a neural network are structured into three layers: the input layer, the hidden layer, and the output layer [23,24,25,26]. One of the most commonly used algorithms in the field of neural networks is the Back Propagation Neural Network (BPNN). BPNN is a type of multilayer feedforward neural network that is trained using the error backpropagation algorithm. This algorithm has gained widespread popularity and is extensively utilized in various applications. Figure 3 shows the architecture of the BP neural network, whose nodes and related connections are illustrated. The output y originates from input x according to the following equation:

(4) $$f\left(x\right)=W^{\left(o\right)}tansig\left(W^{\left(h\right)}*x+b^{\left(h\right)}\right)+b^{\left(o\right)}$$

Here, the superscripts of (o) and (h) mean the output and hidden layers. W is the weight and b is the bias term. Weight means the strength of the connection between the inputs and the output in a linear mode. The training process involves searching for parameter values that minimize a suitable error function. The developed prediction model relies on the creation of a feed-forward type multilevel artificial neural network, featuring one hidden layer with 6–13 neurons. The model’s one output layer represents the sound absorption coefficient of compound samples with JE fibers as cores and the MPPs with face sheets. The raw data altering the parameters of MPPs and JE fibers can be divided into three groups to optimize the artificial models: 70% of raw data with 34,760 samples can be treated as the training set, which can be used to fine-tune the weights according to the error made on the output layers. Then, 15% of them can be applied in the halt training when generalization stops improving, while the rest of the data are for independent measures of network performance during and after training. We used the methods of the Levenberg–Marquardt algorithm (LM algorithm) offered by the toolbox of MATLAB. Compared to the gradient descent, the LM algorithm has a faster convergence speed, especially near the initial point. In this work, based on the designs and structures of MPP-type sound absorbers, parameters of MPPs and JE fillings are used to build related models. The specific ten parameters as output layers are listed in Table S1. 

RMSE (Root Mean Square Error) is defined as the square root of the average of squared differences between the predicted values and the actual values in a regression model. The formula is shown like this:

(5) $$RMSE=\sqrt{\frac{\Sigma {\left(y_{r}-y_{p}\right)}^{2}}{n}}$$

y_r_: the experiment values in this work;

y_p_: the prediction values in this work.

### 2.6. Analytical Model

When sound waves are perpendicularly incident on the surface of sound-absorbing materials, the sound pressure and velocity of particles are continuous. *i*, *r*, and *t* represent incidence, reflection, and transmission, respectively.

(6) $$p_{i}+p_{r}=p_{t}$$

(7) $$u_{i}-u_{r}=u_{t}$$

(8) $$R=\frac{p_{r}}{p_{i}}=\frac{Z-\rho c}{Z+\rho c}=\frac{z-1}{z+1}.$$

(9) $$\alpha_{n}=1-{\left|R\right|}^{2}$$

(10) $$=1-{\left|\frac{z-1}{z+1}\right|}^{2}$$

In this part, *z* represents the surface impedance of materials.

The analytical model of this compound material can be referenced to the electric circuit, where the micro-perforated panel and porous materials can be considered to be connected in series. So, the surface impedance of compound material can be expressed by the following equation:

(11) $$Z_{s}=Z_{s}^{porous}+Z_{s}^{MPP}$$

POROUS PART

For porous materials, when the back plate is rigid, we can use the following equation to calculate the impedance of porous materials:

(12) $$Z_{s}^{porous}=-jZ_{0}^{porous}cotkl$$

(13) $$k=\omega \sqrt{K/\rho }$$

(14) $$Z_{0}^{porous}=\rho_{0}^{porous}c_{0}^{porous}=\sqrt{K\rho }$$

*K* represents the elastic modulus of the porous material, *ρ* denotes the complex density, *c_0_* stands for the complex velocity, and *Z_0_* signifies the characteristic impedance of the materials. To be more specific, the elastic modulus *k* can be expressed by the equation as follows:

(15) $$K=P_{0}[1+\frac{1}{8}(\gamma -1)\frac{j\omega d^{2}}{4v}]$$

where *P_0_*, γ, d, and v represent the air pressure, specific heat ratio of the air, the diameter of the tube (the size of the holes in this work), and effective thermal conductivity of air, respectively.

The complex density of the porous materials can be expressed by the following formula:

(16) $$\rho_{1}=\frac{s_{1}\rho_{0}}{\varphi }[1+{\left(3^{2}+\frac{x_{1}^{2}}{2}\right)}^{-\frac{1}{2}}+\frac{32}{j\omega \rho_{0}d_{1}^{2}}{\left(1+\frac{x_{1}^{2}}{2}\right)}^{\frac{1}{2}}$$

MPP PART

A micro-perforated plate consists of a parallel arrangement of multiple microtubes. In the case of a circular tube, the derivation based on Crandall’s simplified method [27] can be summarized as follows:

(17) $$\rho \dot{\mu }-\frac{\eta }{r_{1}}\frac{\partial }{\partial r_{1}}\left(r_{1}\frac{\partial }{\partial r_{1}}\mu \right)=\frac{\Delta p}{t}$$

where *η* represents the constant dynamic viscosity of air, *μ* denotes the axial particle velocity of the air within the tube (which is a function of the radius vector r1), *t* represents the length of the tube, and Δ*p* indicates the pressure difference between the two ends of the tube. By utilizing Equation (18), the average velocity and specific acoustic impedance of the tube can be calculated.

The solution is the following:

(18) $$\mu \left(r_{1}\right)=-\frac{\Delta p}{\eta \kappa^{2}t}[1-\frac{2}{\kappa r_{0}}\frac{J_{0}(\kappa r_{1})}{J_{0}(\kappa r_{0})}]$$

*Jn* is the n-order Bessel function of the first kind, the definition of *Z* is as follows:

(19) $$Z=\frac{p}{u}$$

(20) $$Z_{1}=\frac{\Delta p}{\bar{\mu }}=\frac{j\omega \rho }{{\left[1-\frac{2}{x\sqrt{-j}}\frac{J_{1}\left(x\sqrt{-j}\right)}{J_{0}\left(x\sqrt{-j}\right)}\right]}^{-1}}$$

(21) $$Z_{s}^{MPP}=\frac{Z_{1}}{P\rho c}=r+j\omega m$$

(22) $$r=\frac{32\mu }{\rho c}\frac{t}{d_{2}^{2}}[\sqrt{1+\frac{x^{2}}{32}}+\frac{\sqrt{2}x}{8}\frac{d_{2}}{t}]$$

(23) $$m=\frac{t}{\rho c}[1+\frac{1}{\sqrt{3^{2}+\frac{x^{2}}{2}}}+0.85]\times \frac{d_{2}}{t}$$

Taking *c* as the speed of sound at 25 °C, the value is 340 m/s. *µ*, dynamic viscosity. The value is 1.56 × $10^{-5}{\;m}^{2}/s$ and the units of *d*, *b*, and *t* are millimeters. The end correction of *0.85 × d/t* is applied because the difference between the perforation diameters and the distance between the perforations are close in size. Other parameters used in this model are listed in Table 1.

### 2.7. Sound Absorption Peak Prediction 

The sound resonance peak *f*_0_ is calculated by Equation (24) [28].

(24) $$f_{0}=\frac{c}{2\pi }\sqrt{\frac{p}{(t+0.8D)L}}$$

*c*: Velocity of sound at a specific temperature;

*p*: The rate of perforation;

*L*: The air space behind the microperforated panel;

*D*: The diameter of the perforated holes;

*t*: Thickness of the face sheet.

### 2.8. The Calculation of the Average Sound Absorption Coefficient and Noise Reduction Coefficient

The average sound absorption coefficient refers to the average value of the sound absorption coefficients at 125, 250, 500, 1000, 2000, and 4000 Hz. This parameter characterizes the material’s ability to absorb sound over a wide frequency range. On the other hand, the noise reduction coefficient (NRC) refers to the average value of the sound absorption coefficients at 250, 500, 1000, and 2000 Hz. It represents the material’s sound absorption ability in the mid-to-low frequency range.

(25) $$Average\;sound\;absorption\;coefficient\;(ASAC)=\frac{{SAC}_{125Hz}+{SAC}_{250Hz}+{SAC}_{500Hz}+{SAC}_{1000Hz}+{SAC}_{2000Hz}+{SAC}_{4000Hz}}{6}$$

(26) $$NRC=\frac{{SAC}_{250Hz}+{SAC}_{500Hz}+{SAC}_{1000Hz}+{SAC}_{2000Hz}}{4}$$

*SAC* means the sound absorption coefficient at a specific frequency.

## 3. Results and Discussion

**Figure 4:**
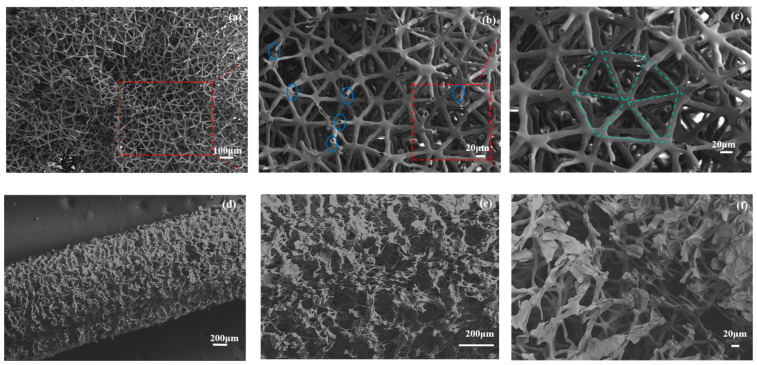
SEM images of the cross-sectional (**a**–**c**) and longitudinal sectional (**d**–**f**) morphology for JE fibers.

Figure 4 shows the SEM micrographs of JE fibers with different sections. Figure 4a–c illustrates the cross-sectional direction. The structure exhibited by JF fibers consists of a hexagonal network of microfibers. More interestingly, the elementary units are not located in the same plane and they form a complex three-dimensional porous structure. The repeating element was essentially triangular with a side length of 60 µm. Figure 4b illustrates that the elementary fibers are hollow structures (annotated in blue), contributing to their lightweight efficiency. The diameter of the elementary fiber was approximately 5 μm. The green dotted lines show the elementary hexagonal skeleton of JE fibers. Figure 4d–f shows the longitudinal view of the single JE fiber. Compared with the common morphology of natural fibers [7,18], the morphology of JE fibers is intricate, featuring hierarchical pores and channels, which suggests a strong potential for efficient sound energy dissipation.

**Figure 5:**
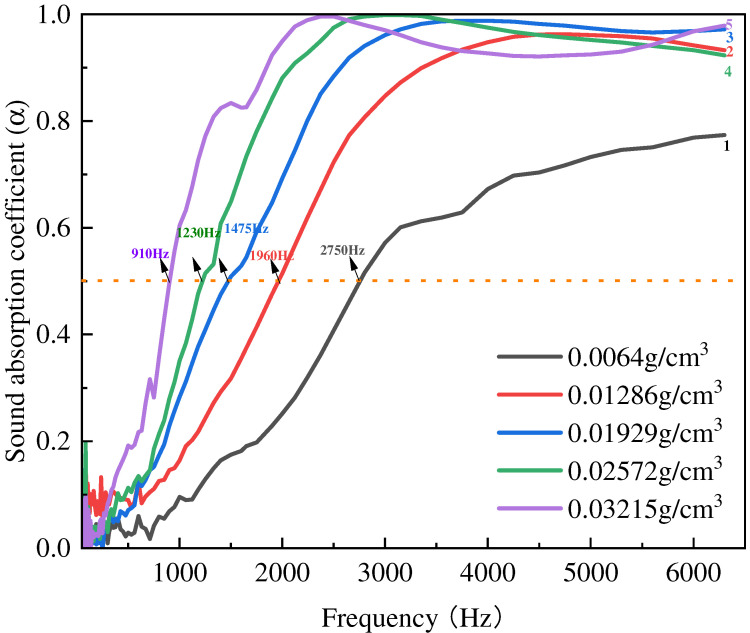
The sound absorption coefficient of JE fibers with different filling densities.

Firstly, the sound absorption performance of JE fibers was researched. The true density of JE fibers was measured at 0.36 g/cm^3^. Figure 5 shows that the sound absorption of JE improved with denser samples. As density increased, the peak absorptions occurred at a lower frequency and the maximum value increased from 0.77 to 0.98. As the density increases, the peak values remain close to one, while the frequency range of the sound absorption peak decreases. The NRC and average sound absorption coefficients are listed in Table 2. The sound energy lost due to the increased complexity of the sound path (tortuosity) in an absorber is directly proportional to the density of the absorber material. In other words, as the density increases, more energy is lost in the absorber [24,25].

**Figure 6:**
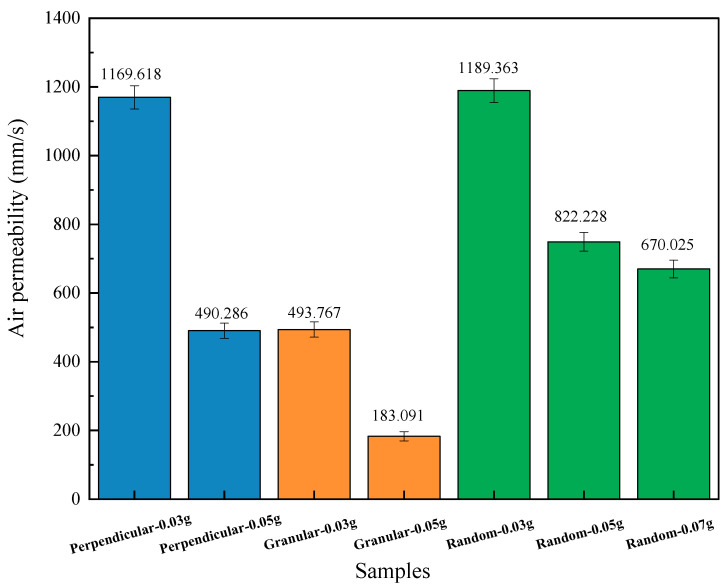
The air permeability of honeycomb with different JE filling densities and geometric arrangement fibers as fillings.

The air permeability results of honeycombs with different JE densities and geometric arrangements are presented in Figure 6. The filling density and geometric arrangement strongly influenced air permeability. It is found that a higher JE density resulted in lower air permeability since the denser fillers prevent air from flowing through the JE/honeycomb cell. [29] With the same JE filler density, the air permeability is presented as random>perpendicular>granular in this work.

**Figure 7:**
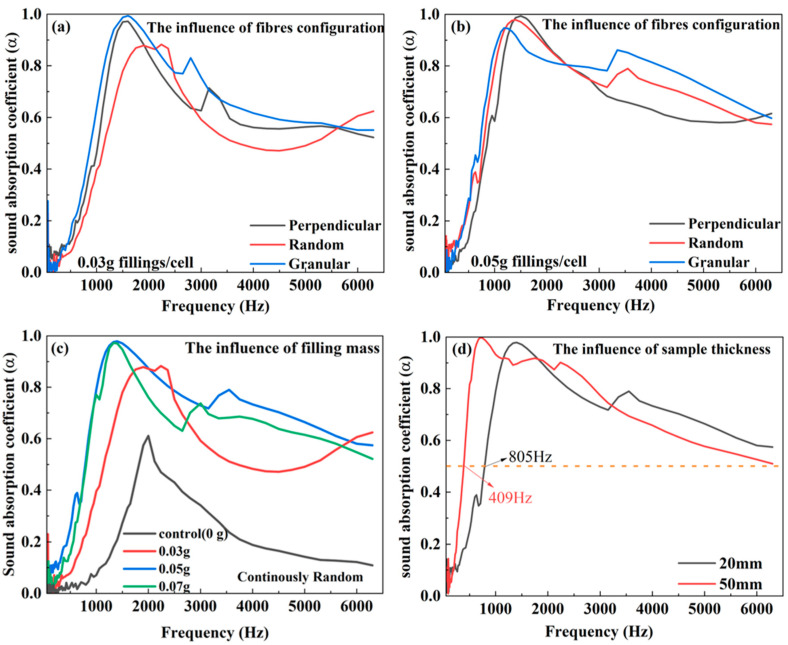
The effects of different configurations of JE as fillings (**a**,**b**); the effects of different filling masses of JE as fillings (**c**); the effects of different thicknesses of JE as fillings (the MPP fixed at 1 mm perforation diameter and 13% perforation rate) (**d**).

The sound absorption performance of MPP-type honeycomb with different geometrical arrangements of JE fiber fillings with the same filling density is shown in Figure 7a,b. The granular samples perform best among these samples, possibly due to the lowest value of air permeability caused by the most complex tortuosity with smaller particles. The random samples perform worst because of the highest value of air permeability, affecting less acoustic energy dissipation. This indicates that in these structures with MPP, honeycomb, and porous fillings, the average sound absorption coefficient may be closely related to the air permeability of the filled materials. According to the data from these two groups, the group with the lowest air permeability had the best sound-absorbing effects. Figure 7c compares the effects of the filling density of JE fibers on the sound absorption of MPP-type absorbers with a continuous random arrangement. Increasing filling density in honeycomb cells may enhance the sound absorption performance but the largest density adversely affects the sound absorption. When the fibers in the absorber are packed too densely, a reversed trend is expected. This is because the porosity of the absorber decreases, which in turn restricts the penetration of sound waves into the absorber [30,31]. Figure 7d shows the sound absorption performance of random JE fibers in honeycombs with different thicknesses. The sound absorption coefficients of samples with different mass fillings are listed in Table 3. The sound absorption coefficient at some specific frequency of samples with different thicknesses is listed in Table 4 Sample thickness was previously noted as the most critical parameter to influence sound absorption [32]. In this work, the absorption peak of 50 mm-thickness samples is located at 740 Hz, whereas the peak for thinner samples increased to 1400 Hz. The high-frequency absorption of 20 mm and 50 mm samples decreases because of the MPP face sheets.

**Figure 8:**
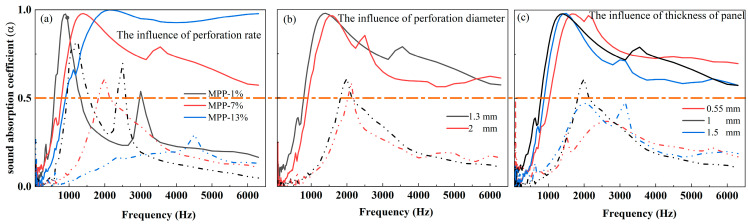
The effects of the parameters of MPP on the sound absorption performance of samples (**a**); the effects of the perforation rate on the sound absorption performance of filled samples (**b**); the effects of the perforation diameter on the sound absorption performance of the filled samples (**c**); the effects of the thickness of microperforated panels on the sound absorption of filled samples. (The solid lines illustrate the acoustic performance of filled samples, while the dotted lines represent the corresponding characteristics of empty cells).

The characteristics of the sound absorption curves of MPP-type silencers are similar, with three stages evident: the primary, middle, and stable stage. It is recommended that the point of demarcation is 0.5 (shown with dotted orange lines in Figure 8), which corresponds to the sound absorption coefficient of the initial size [33,34]. The sound absorption coefficient at some specific frequency of samples with different MPPs is listed in Table 5. When the absorption coefficient is 0.5, it suggests that the material absorbs half of the energy of the sound waves at a specific frequency while reflecting the other half. This value can be considered as a reference point for comparing the sound absorption abilities of different materials at a particular frequency. The fillings of JE fibers thus improved the acoustic attenuation of the MPP-type honeycomb structures, enhancing the peak value and broadening the absorption range. Figure 8a shows the influence of the perforation rate of MPP with 0.05g/cell in a random JE in a honeycomb. The fillings of JE fibers significantly improved the sound absorption of samples. Figure 8a shows the effects of the perforation rates (1%, 7%, and 13%) on the sound absorption performance. According to Equation (22), their sound absorption peak should be located at 850 Hz, 2200 Hz, and 3050 Hz, respectively, while the fillings of JE enhance the frequency range of MPP and move them to a lower frequency range. The sound absorption of 7% of samples performed best, with an average sound absorption coefficient of 0.477. The samples at a 13% perforation rate performed similarly to the porous materials, exhibiting a stable level of around 0.9 beyond 2100 Hz. Figure 8b illustrates the effect of perforation diameter on sound absorption. The performance of two unfilled structures was similar and the corresponding properties of filled samples illustrate similar trends. Therefore, a slightly larger perforation size can be selected for more convenient manufacturing. The plate thickness dramatically influenced the sound absorption peak when empty but had a slight influence on the filled structure. However, it only marginally influenced the absorption peak’s position and, therefore, the benefit of face sheet thickening remains uncertain. The influence of these parameters on the sound absorption coefficient is as follows: air gap depth > perforation diameter > perforation rate > panel thickness [18,35].

**Figure 9:**
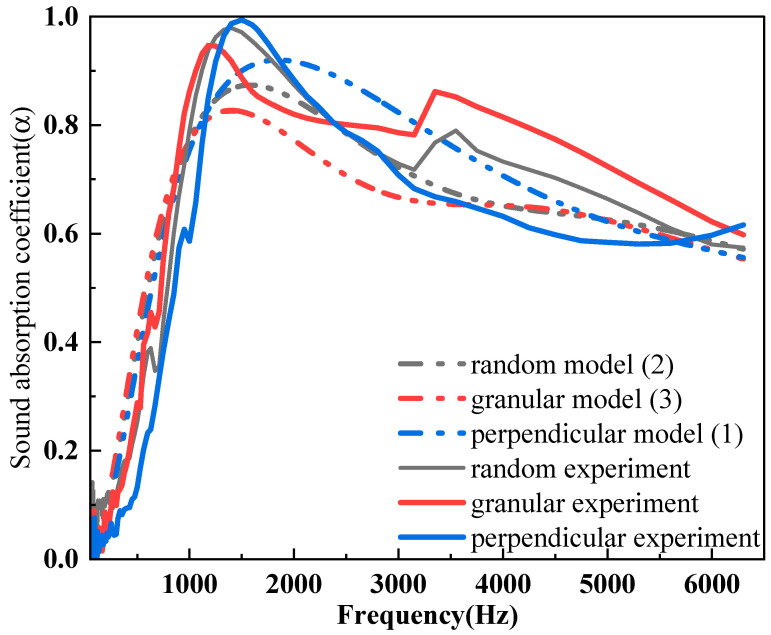
The comparison of analytical and experimental models of 0.05 g samples.

Figure 9 shows the analytical and experimental sound absorption curves of samples filled with 0.05 g of JE fibers in one cell of honeycomb. The porosity and density of these three kinds of JE fibers are similar and their sound absorption performance depends on the tortuosity mentioned in the Johnson–Champoux–Allard (JCA) semi-empirical models [4,36]. Generally, the structural factors of porous acoustic absorbers are taken from 2–10 according to their tortuosity [28]. Some suggest that the tortuosity can range from 1–3 [6,37]. Figure 9 further illustrates the potential importance of tortuosity. The perpendicular, random, and granular samples are, respectively, taken with 1, 2, and 3 as structural parameters to predict sound absorption performance in this analytical model. The analytical models are conducted in MATLAB and the related parameters used in this model are shown in Table 1; the numerical predictions for 0.05 g of JE fibers filled in the honeycomb are shown in Figure 9 against the corresponding experimental results. The RMES of numerical analytical results reflect that the sound absorption characteristics of perpendicular, random, and granular arrangements are 0.0674, 0.0504, and 0.0727, respectively. The prediction of first peak positions is relatively accurate. Since the theoretical model ignores the influence of the structure’s natural resonance (the structures are regarded as a rigid boundary), the theoretical calculation does not reflect the peak at 3500 Hz of the experimental sound absorption curve [38]. Additionally, the magnitude of peak absorption, curve trends, and low-frequency absorption performance are predicted relatively accurately. The structural factor can affect the acoustic performance of the material, especially the sound absorption effect in the high-frequency range [28]. Materials with more complex and evenly distributed pores typically exhibit better sound absorption performance, as they can effectively dissipate sound waves and reduce the reflection of sound energy.

**Figure 10:**
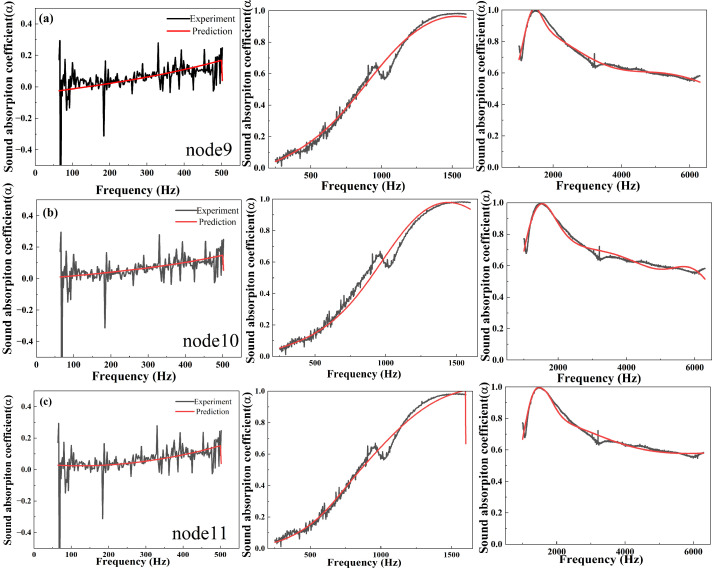
(**a**–**c**) The comparison of experiments and predictions from machine learning of perpendicular samples with 0.05 g JE fibers in one cell.

As shown in Figure 10, for perpendicular 0.05 g samples with 20 millimeters thicknesses, the sound absorption coefficients measured at the low-frequency range are unstable because the low-frequency attenuation is easily influenced by the vibration and disturbance, while fixing curves are relatively smooth. The simulated curves strongly correlate with the absorption coefficient data obtained via impedance tube measurements for normal incidence. For the medium frequency range of 250–1600 Hz, compared with the experiment results, the small sound absorption peak is not fitted by the machine learning models, while the trends at the high-frequency range (1000–6300 Hz) closely match the measured absorption coefficient data. According to the prediction data based on nodes 9, 10, and 11, the RMSE values of them are 0.0466, 0.0453, and 0.0410, respectively, showing the accuracy of machine learning models.

**Figure 11:**
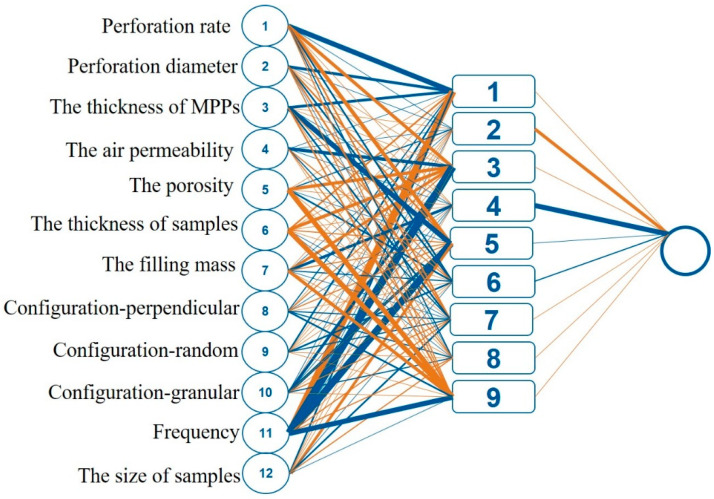
The weights and bias of 12 input parameters in the artificial neural network model architecture with three layers. (9 nodes).

The thickness of the lines connecting neurons between different levels indicates the connection’s weight: the thicker the line, the greater the weight. The connections between the input and hidden layer are schematically illustrated in Figure 11, which shows the importance of input parameters. The blue and orange lines respectively represent positive and negative impacts. The detailed values of weight and bias are listed in Tables S2 and S3. As shown in Figure 11, the top three input parameters are the frequency, porosity, and thickness of samples, consistent with our previous experience [18]. It can be roughly speculated that the most influenceable factors are the filling mass, frequency, and thickness of samples. The related code to predict the sound absorption performance is attached in Supplementary Materials.

For numerical models, the prediction accuracy of them is not as good as machine learning models. However, we do not need much data to realize the prediction functions; efficiency is high. We can know how the variables influence the sound absorption coefficients to some extent. For machine learning models, the prediction accuracy is quite decent with lower RMSE but we need a large number of data to train the models. In addition, the weights of connections can partly reflect the importance of the input parameter. However, we cannot know the mechanism behind the samples.

**Figure 12:**
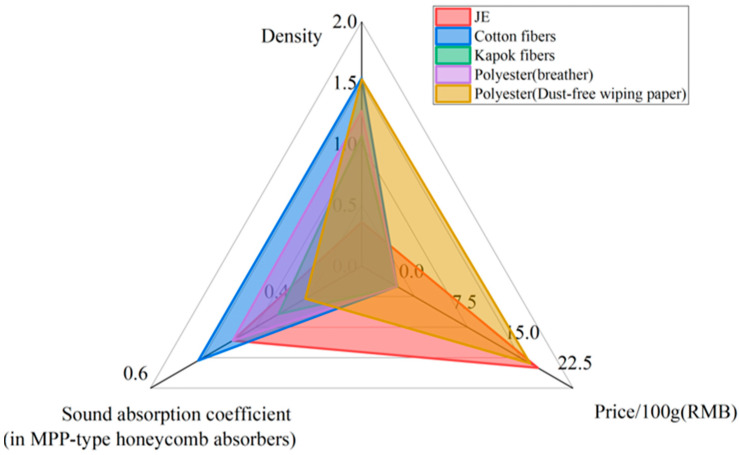
Radar charts of different fibers to compare the density, price, and acoustic attenuation ability (the colorful lines represent the filled honeycomb samples with different natural fibers, while the black line shows unfilled honeycomb cell results).

Table 6 demonstrates the parameters that influenced the sound absorption of acoustic absorbers researched in this work, which consist of the MPP and porous absorbers connected in series. This table not only outlines the impact of these parameters on individual absorbers but also provides an overview of their effects on composite absorbers. It can be observed that the influence of these parameters on individual absorbers is similar to their impact on composite absorbers. Figure 12 compares the sound absorption ability, density, and approximate cost of the different solutions. We can see that JE fibers are competitive in the fields of lightweight and performance compared with other natural fibers.

## 4. Conclusions

JE fibers were considered a promising sound-absorbing material, usable either independently as porous sound-absorbing materials or as fillers in combination with microperforated panels and honeycomb structures. By utilizing both an analytical model and an artificial neural network (ANN) model, the sound absorption characteristics of these panels were accurately predicted. The results indicate that JE fibers significantly enhance the acoustic performance of honeycomb sandwich panels, making them competitive with other natural fibers in terms of lightweight and performance.

Our findings indicate that JE fibers, when incorporated into honeycomb structures, significantly enhance sound absorption capabilities while maintaining a lightweight profile. This finding is particularly evident when comparing the sound absorption, density, and cost-effectiveness of JE fibers against other natural fibers. This highlights that JE fibers are competitive and advantageous in performance and weight, making them a viable option for acoustic applications. In summary, JE fibers exhibit promising potential as an effective and lightweight solution for sound absorption in honeycomb sandwich panels. Future research could explore optimizing fiber distribution and integrating these findings into commercial acoustic products.

Natural fibers exhibit several drawbacks, including a substantial diameter, inadequate moisture resistance, poor antifungal properties, low fire retardancy, and insufficient fiber–matrix adhesion, potentially restricting their commercial utility. More efficient pretreatment methods with low environmental impact are meaningful in accelerating natural fiber usage in the building and automobile industries. Last but not least, involving the Life Cycle Assessment in natural fiber sound absorber development will benefit the long-term sustainable growth of natural fiber applications.

## Figures and Tables

**Table 1:** The parameters of theoretical models to predict the sound absorption coefficients.

Property	Value
Air density, ρ_0_ (Kg/m^3^)	1.295
Atmosphere pressure, P_0_ (Pa)	1.01 × 10^5^
Dynamic viscosity of air, η	1.85 × 10^−5^
Specific heat ratio of air, γ	1.4
Perforation ratio of the perforated plate, p_1_	0.07
Porosity of JE fibers (0.05g/cell), φ	0.96
Thickness of the micro-perforated plate, t_1_ (mm)	1
Thickness of the porous material, *l* (m)	0.02
Diameter of the perforation, d_2_ (mm)	1.3

**Table 2:** Sound absorption coefficient at the specific frequencies and the average value of JE fibers.

Filling Density (g/cm^3^)	Porosity	125 Hz	250 Hz	500 Hz	1000 Hz	2000 Hz	4000 Hz	ASAC	NRC
0.0064	0.98	0.02	0.026	0.029	0.096	0.252	0.673	0.122	0.10
0.01286	0.96	0.065	0.054	0.09	0.164	0.515	0.948	0.306	0.20
0.01929	0.95	0.04	0	0.071	0.283	0.693	0.988	0.346	0.30
0.02572	0.93	0.043	0.048	0.113	0.351	0.881	0.975	0.402	0.35
0.03215	0.91	0	0.028	0.192	0.604	0.95	0.927	0.450	0.45

**Table 3:** Sound absorption coefficient at the special frequencies and the average value of samples with JE fibers as fillings.

Mass in One Cell	Porosity	Arrangement	125 Hz	250 Hz	500 Hz	1000 Hz	2000 Hz	4000 Hz	ASAC	NRC
0.03g	0.98	Granular	0.038	0.029	0.149	0.634	0.905	0.617	0.395	0.45
		Perpendicular	0.099	0.078	0.119	0.462	0.845	0.561	0.361	0.40
		Random	0.024	0.031	0.079	0.396	0.872	0.483	0.314	0.30
0.05g	0.96	Granular	0.015	0.097	0.288	0.862	0.82	0.815	0.483	0.50
		Perpendicular	0.027	0.066	0.133	0.586	0.883	0.632	0.388	0.40
		Random	0.108	0.103	0.253	0.791	0.874	0.733	0.477	0.50
0.07g	0.95	Random	0.048	0.07	0.154	0.771	0.761	0.677	0.413	0.45

**Table 4:** Sound absorption coefficients at the specific frequencies of samples with different thickness.

Details of Fillings	JE Thickness	125 Hz	250 Hz	500 Hz	1000 Hz	2000 Hz	4000 Hz	ASAC	NRC
0.05g/cell	20 mm	0.108	0.103	0.253	0.791	0.874	0.733	0.477	0.50
0.125g/cell	50 mm	0.068	0.196	0.817	0.932	0.894	0.658	0.594	0.70

**Table 5:** Sound absorption coefficient at the specific frequencies and the average value of JE absorbers.

Perforation Diameter	Thickness of Panel	Perforation Rate	125 Hz	250 Hz	500 Hz	1000 Hz	2000 Hz	4000 Hz	ASAC	NRC
1.3 mm	1 mm	1%	0.06	0.083	0.281	0.876	0.274	0.227	0.300	0.40
	1 mm	7%	0.108	0.103	0.253	0.791	0.874	0.733	0.477	0.50
	1 mm	13%	0.015	0.058	0.13	0.602	0.994	0.927	0.454	0.45
2 mm	1 mm	7%	0.029	0.023	0.119	0.619	0.846	0.595	0.372	0.40
1.3 mm	0.55 mm	7%	0.01	0.04	0.121	0.464	0.934	0.726	0.383	0.40
	1.5 mm	7%	0.007	0.029	0.119	0.674	0.836	0.607	0.379	0.40

**Table 6:** Various parameters affecting the acoustic performance of absorbers; (a) the comparison of porous absorbers [28] and MPP-type honeycomb absorbers in this work and (b) the comparison of MPP absorbers [28] and MPP-type honeycomb absorbers in this work.

(a) Factors Influencing the Sound Absorption Performance of Porous Materials/MPP-type honeycomb absorbers	The details of how to influence the sound absorption coefficient of the porous absorbers in reference	The details of how to influence the sound absorption coefficient of MPP-type acoustic absorbers
Air permeability	Optimal Air Permeability for Improving the Sound Absorption of Porous Materials	Similar impact trends on porous materials
Porosity	The porosity of porous materials is generally over 90% and the porosity of dense materials is low, which is not beneficial to the sound absorption performance	The values of porosity are high in this work. According to machine learning models, porosity may be the second most influential factor among these variables
Tortuosity (structural factor and arrangements)	Structural factors have less influence on low-frequency sounds. When the air permeability is high, increasing the structural factor leads to periodic changes in the sound absorption coefficient of the material within the mid- to high-frequency range	This parameter is difficult to test and visualize. It can be verified through the numerical models
Sample thickness	The enhanced thickness of samples will increase low-frequency absorption. Peak absorption occurs at the resonant frequency of one-quarter of the wavelength of the incident sound	An increase in thickness will shift the peak to lower frequencies and the absorption peak width will increase The presence of MPP shifts the absorption peak toward lower frequencies
Density of samples	Changing the bulk density will first cause the middle and high absorption change	An optimal density exists in this work
(b) Factors Influencing the Sound Absorption Performance of MPP/MPP-type honeycomb absorbers	The details of how to influence the sound absorption coefficient of porous absorbers in reference	The details of how to influence the sound absorption coefficient of MPP-type acoustic absorbers
The perforation rate	The lower perforation rate of MPP will move the absorption peak to a lower frequency range	Filling provides less enhancement for low perforation yet more enhancement for higher perforation range
The perforation diameter	Reducing the pore size is equivalent to decreasing the perforation rate, causing the absorption peak to shift toward lower frequencies	The pore size difference in this work is not significant, so the sound absorption curves look similar
The panel thickness	An enhancement in thickness will result in a slight shift in the peak toward the lower frequency range	The sound absorption performance does not change significantly in this work

## Data Availability

Data are contained within the article. Data available in a publicly accessible repository.

## Supplementary Materials

The following supporting information can be downloaded at: https://www.mdpi.com/article/10.3390/polym16131953/s1, Figure S1: the photographs of samples based on different arrangements and densities of JE fibres (a) show the random JE fibres in honeycomb cell (the mass in one cell from top to bottom are 0.07 g, 0.05 g and 0.03 g); (b) illustrate the perpendicular particles, 0.05 g/cell (up) and 0.03 g/cell (down); (c) present the granular particles from up 0.05 g/cell to down0.03 g/cell; Figure S2: the photographs of microperforation panels used in this work, (a–c) show the different perforation rate of panels; (d–f) illustrate the thickness of panels; (g) represent panels with the perforation diameter of 2 mm; Table S1: the parameters of samples used in ANN models; Table S2: Best weights and biases returned by the model (Input layer to Hidden layer); Table S3: Best weights and biases returned by the model (Hidden layer to Output layer).
