# Supplementary material for: Sound Absorption Performance of Ultralight Honeycomb Sandwich Panels Filled with “Network” Fibers—Juncus effusus
Source: Polymers (Basel). 2024 Jul 8;16(13):1953. doi: 10.3390/polym16131953 (PMC11244036; doi:10.3390/polym16131953)
Supplement: Supplementary file 1 [file polymers-16-01953-s001.zip › polymers-3059647-supplementary.pdf]

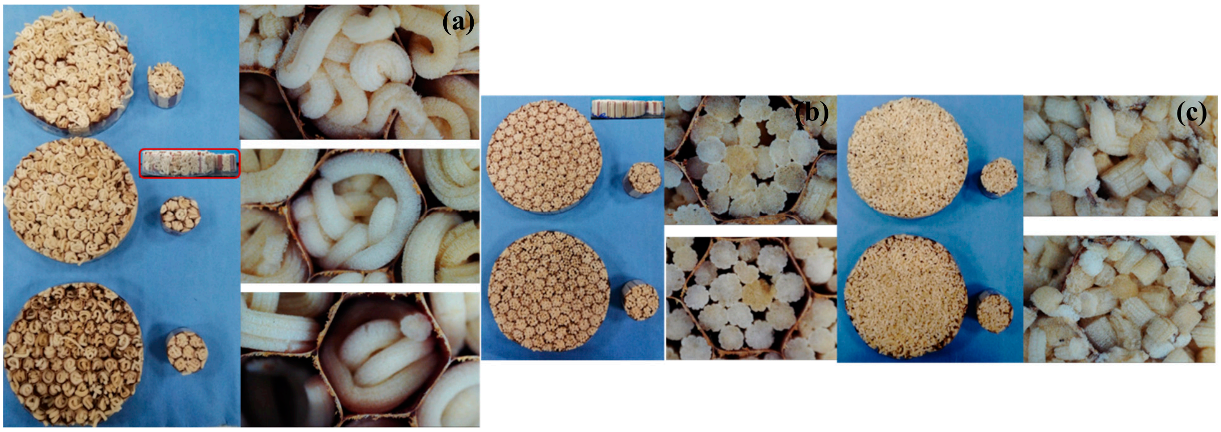

Figure S1. the photographs of samples based on different arrangements and densities of JE fibres (a) show the random JE fibres in honeycomb cell (the mass in one cell from top to bottom are 0.07g, 0.05g and 0.03g); (b) illustrate the perpendicular particles, 0.05g/cell (up) and 0.03g/cell (down); (c) present the granular particles from up 0.05g/cell to down0.03g/cell.

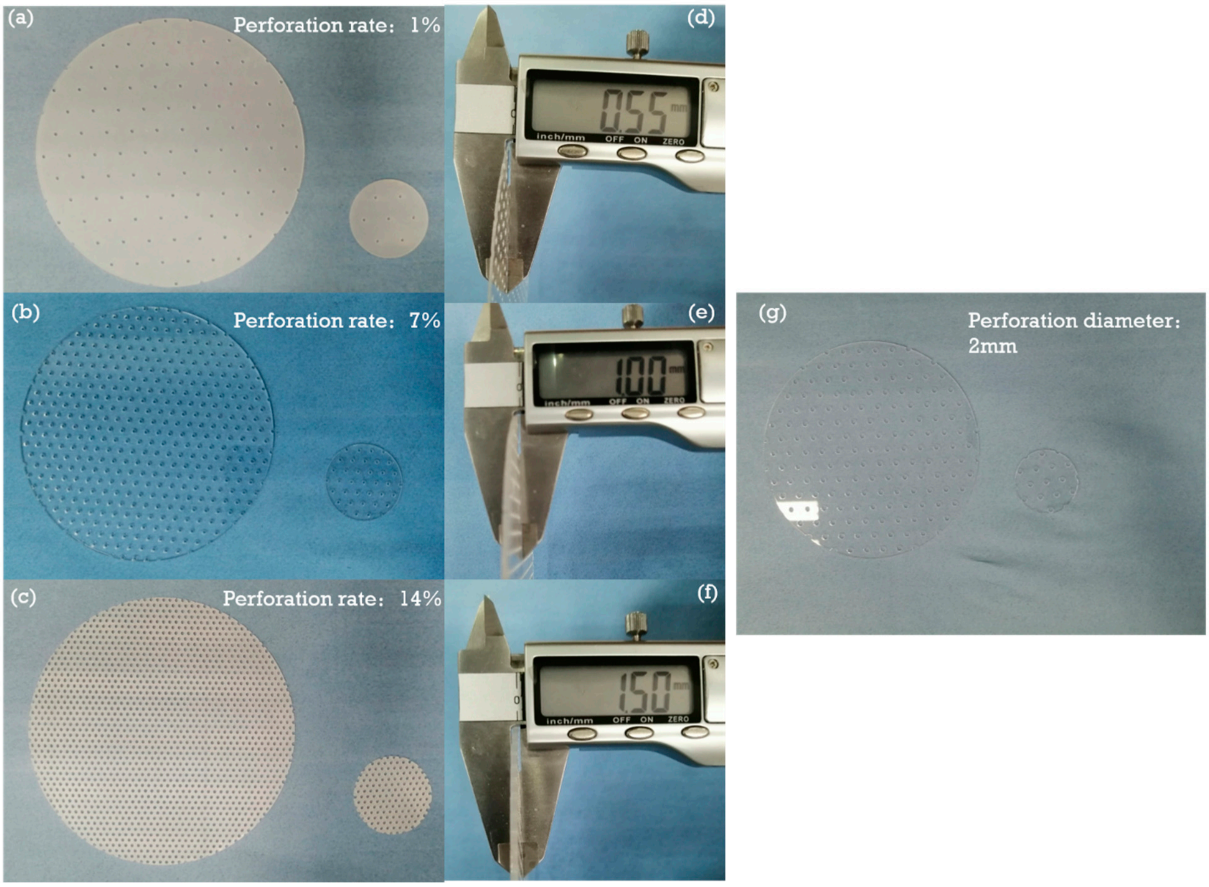

Figure S2. the photographs of microperforation panels used in this work, (a), (b), and (c)show the different perforation rate of panels; (d), (e) and (f) illustrate the thickness of panels; (g) represent panels with the perforation diameter of 2mm

Table S1. The parameters of samples used in ANN models

Components	Parameters	Setting variables
**The variables of MPP**	The effects of perforation rate	1%<br>7%<br>13%
	The effects of perforation diameter	1.3mm<br>2mm
	The effects of panel thickness	0.55mm<br>1mm<br>1.5mm
**The variables of JE fillings**	The effects of density (filling mass)	0.03g<br>0.05g<br>0.07g
	The effects of configuration	Non-continuously

		Perpendicular
		Continuously random
		Non-continuously
		Granular
	The effects of thickness of JE fillings	20mm
		50mm
	Porosity (depends on filling mass and true density)	0.95
		0.96
		0.98
	Air permeability	Shown in figure 4
**Compound absorbers**	The size of the samples	3cm
		10cm
	Frequency range	63-6300Hz

Table S2. Best weights and biases returned by the model (Input layer to Hidden layer).

	H1	H2	H3	H4	H5	H6	H7	H8	H9
I1	3.770676	0.025963	-2.2735	-0.33248	-4.82216	1.043421	-2.23192	-0.30305	-1.0592
I2	0.823443	0.00397	-0.59594	-0.20392	-1.85919	1.339008	-2.09306	0.686639	-1.88197
I3	1.556931	-0.00152	-0.24209	0.331849	8.458161	-0.89536	2.121959	-0.61285	-2.96292
I4	0.476623	0.081749	2.452639	0.514272	0.593207	-1.00374	1.04975	-1.37259	-0.85946
I5	-0.04279	0.16731	-3.25105	-0.2419	-1.54655	2.382863	-0.52612	-0.35616	-6.63588
I6	-1.78528	-0.72552	-3.29237	-0.93553	-1.32318	-0.39989	0.477643	0.374756	-5.1197
I7	-0.24847	0.795145	-1.74125	0.966694	-2.24247	1.537035	0.852941	-0.42059	-2.10835
I8	1.039192	-0.26112	-0.44474	0.163378	-1.21675	-0.70164	1.436192	1.343346	1.880183
I9	2.722188	-0.42926	-1.88036	-0.49327	1.694052	1.184947	0.360564	-0.65876	-0.12861
I10	1.089276	-0.26151	1.139553	0.290218	-0.54474	-0.87786	-1.57505	1.582134	-0.13727
I11	-7.2769	0.350532	10.40782	0.220849	8.021904	-0.07757	-1.86126	-1.71992	5.129892
I12	1.712599	-0.64082	-0.3444	-0.47954	-2.64134	0.305477	2.037442	-1.38118	0.644377
I13	3.770676	0.025963	-2.2735	-0.33248	-4.82216	1.043421	-2.23192	-0.30305	-1.0592

Table S3. Best weights and biases returned by the model (Hidden layer to Output layer).

	H1	H2	H3	H4	H5	H6	H7	H8	H9
O	-0.19067	-2.37534	-0.10374	3.074311	0.218986	1.171267	-0.15599	-0.35889	0.045353
